# Improved lithium-ion battery anode capacity with a network of easily fabricated spindle-like carbon nanofibers

**DOI:** 10.3762/bjnano.7.120

**Published:** 2016-09-14

**Authors:** Mengting Liu, Wenhe Xie, Lili Gu, Tianfeng Qin, Xiaoyi Hou, Deyan He

**Affiliations:** 1 School of Physical Science and Technology, Key Laboratory for Magnetism and Magnetic Materials of the Ministry of Education, Lanzhou University, Lanzhou 730000, China

**Keywords:** carbon nanofiber network, electrospinning, lithium-ion battery, manganese oxide, nitrogen modification

## Abstract

A novel network of spindle-like carbon nanofibers was fabricated via a simplified synthesis involving electrospinning followed by preoxidation in air and postcarbonization in Ar. Not only was the as-obtained carbon network comprised of beads of spindle-like nanofibers but the cubic MnO phase and N elements were successfully anchored into the amorphous carbon matrix. When directly used as a binder-free anode for lithium-ion batteries, the network showed excellent electrochemical performance with high capacity, good rate capacity and reliable cycling stability. Under a current density of 0.2 A g^−1^, it delivered a high reversible capacity of 875.5 mAh g^−1^ after 200 cycles and 1005.5 mAh g^−1^ after 250 cycles with a significant coulombic efficiency of 99.5%.

## Introduction

Lithium-ion batteries (LIBs) have been identified as one of the most advanced inventions in high energy density storage devices and are extensively utilized in various electronic systems for mobile phones, computers and vehicles [1–3]. It is common knowledge that the capacity and energy density of LIBs are highly dependent on the electrode materials. Commercial graphite, with low specific capacity and poor rate capability, no longer meets the urgent requirements of modern technologies as an anode material for LIBs [4–5]. Hence, exploring new candidates with higher energy density and better cycling endurance becomes imperative.

Presently, transition metal oxides are the focus of intensive efforts for LIB anode materials due to their remarkable specific capacity, low cost and environmental compatibility [6–11]. Manganese oxide (MnO) is a particularly good choice owing to its high theoretical specific capacity of 755 mAh g^−1^, low conversion potential and small voltage hysteresis [8,12–13]. However, as a typical perovskite semiconductor, MnO usually suffers inherently poor conductivity and severe volume change during Li^+^ insertion/extraction, which can seriously lead to capacity fading and further limit its application [4–5,8]. Encouragingly, Liu et al. reported a high capacity of 797.6 mAh g^−1^ at 0.1 A g^−1^ by embedding MnO nanoparticles in carbon microsheets [14]. Zhao et al. prepared a composite of MnO and reduced graphene with a capacity of 900 mAh g^−1^ at 0.1 A g^−1^ [7]. Cui et al. obtained a capacity of 841 mAh g^−1^ at 0.1 A g^−1^ by combing pompon-like MnO nanocrystallites with carbon nanotube scaffolds [12]. Wang et al. and Zhao et al. claimed that the electrospun MnO–C composite nanofibers preformed high reversible capacities of 663 and 1082 mAh g^−1^, respectively, at a current density of 0.1 A g^−1^ [5,13]. On the whole, hybridizing MnO and carbon materials can effectively resolve problems such as the high conductivity of the carbon materials and allows a buffering for the volume expansion or contraction to some degree. Moreover, many studies have shown that N modification can improve the conductivity and Li^+^ storage performance of carbon materials [4,8,15]. Based on these pioneer works, the electrochemical properties of a system consisting of carbon materials, MnO nanostructures and a N element should be further studied for the development of high-performance LIBs.

Various MnO–C composites with controlled nanostructures have been synthesized for LIB electrodes to date [4,8,14,16–21]. However, most of the synthesis strategies were two-step routes, combing hydrothermal reaction and other post-treatments. Additionally, the nanostructures synthesized by hydrothermal processes are highly sensitive to the reaction parameters and difficult to repeat. At the same time, the route of electrospinning followed by heat treatment has received significant attention because of its simplicity, high efficiency and versatility for preparation of diverse one-dimensional structures [5,13,22–23].

In this work, a network of spindle-like carbon nanofibers anchored with MnO and N for LIB anodes was fabricated via a simplified synthesis route involving electrospinning followed by preoxidation in air and postcarbonization in Ar. The microstructure, chemical composition and electrochemical lithium storage performance were investigated in detail. As a binder-free LIB anode, the network impressively delivered a high reversible capacity of 875.5 mAh g^−1^ after 200 cycles and 1005.5 mAh g^−1^ after 250 cycles with a coulombic efficiency of more than 99.5% at a current density of 0.2 A g^−1^.

## Results and Discussion

**Figure 1:**
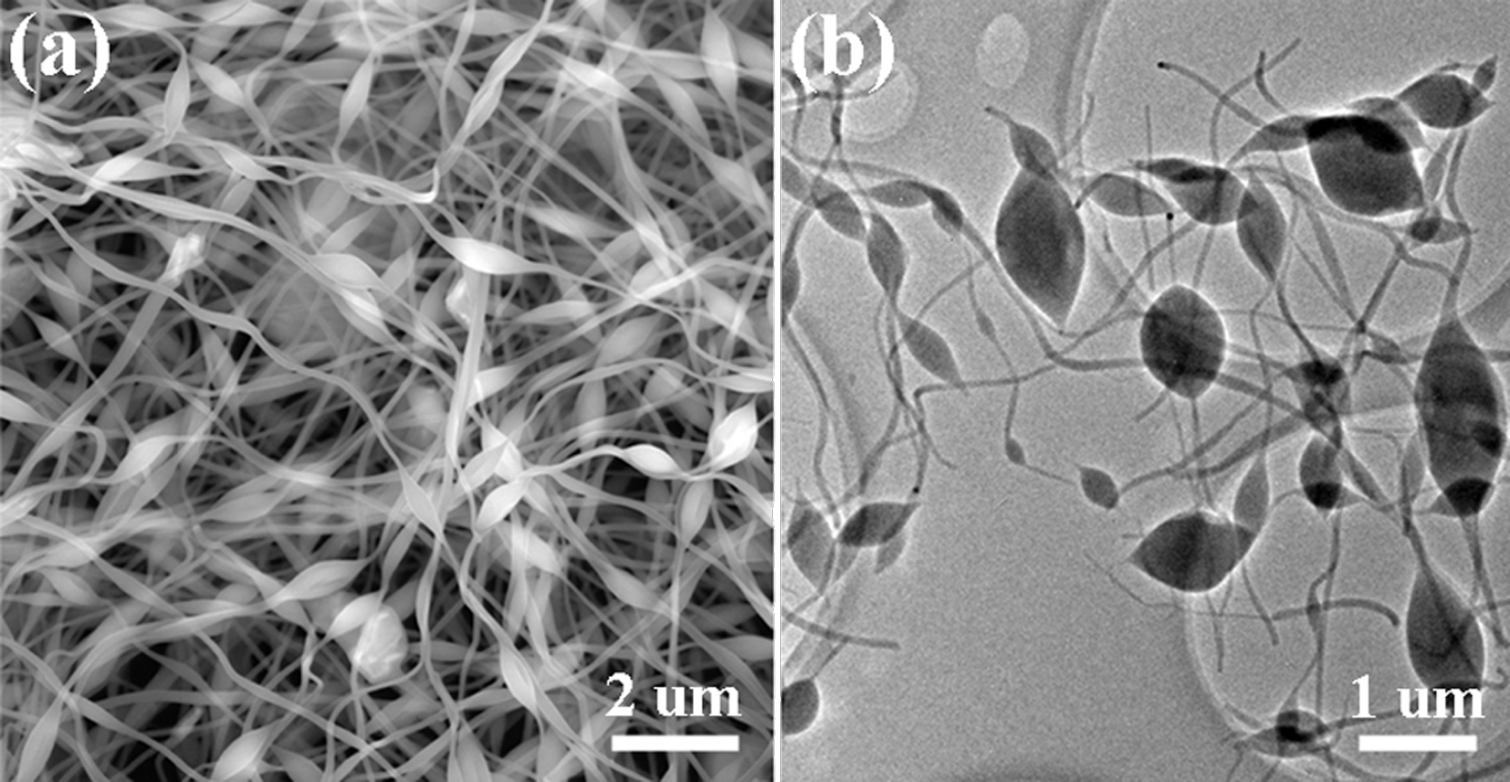
(a) SEM and (b) TEM images of the network of spindle-like carbon nanofibers anchored with MnO and N.

Figure 1a shows a typical SEM image of the as-fabricated nework, which consists of an abundance of beaded nanofibers with a smooth surface, random orientation and several micrometers in length. The unique morphology of the nanofibers is characterized by connected spindle-like beads with ultrafine fibers connected one-by-one along the long axis. The cross-sectional diameter of the beads and fibers are in the range of 350–400 nm and 80–100 nm, respectively. The observation derived from the TEM image shown in Figure 1b verifies the SEM image results. Earlier reports have indicated that the formation of the spindle-like beads on the electrospun nanofibers depends mainly on the viscosity and surface tension of the spinning solution, spinning voltage and receving distance [24–25]. Viewed as a whole, these beaded nanofibers are closely entangled with each other and develop a robust multilayer network, which can be directly used as a binder-free LIB eletrode. Additionally, lithium ions can insert adequately into the network through crevasses between these interlaced nanofibers. Hence, the novel network makes a good LIB electrode.

**Figure 2:**
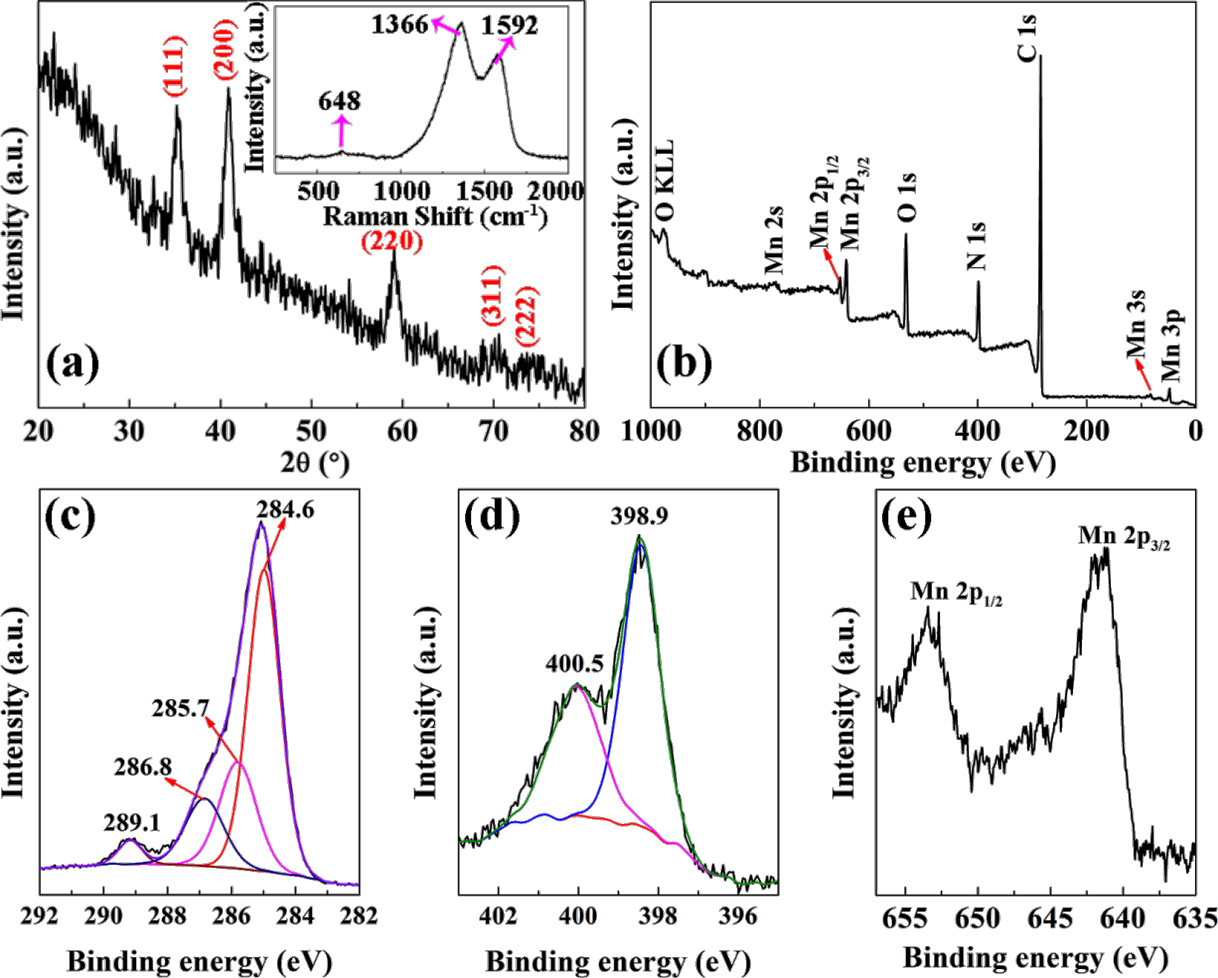
(a) XRD pattern and Raman spectrum (inset), (b) full scan XPS spectrum, (c–e) high-resolution XPS spectra of C 1s, N 1s, and Mn 2p of the spindle-like carbon nanofibers anchored with MnO and N.

Figure 2a displays the XRD pattern of the sample. The distinct diffraction peaks at 35.2°, 40.8°, 59.0°, 69.9° and 73.4° can be respectively assigned to the (111), (200), (220), (311) and (222) planes of cubic MnO (JCPDS 07-0230) [5]. The broad peaks with weak intensity indicate that a low content of nanocrystalline MnO is found in the sample. No obvious carbon signals could be observed in the XRD pattern, meaning that the carbon nanofibers derived from the carbonization of the polyacrylonitrile (PAN) precursor has an amorphous structure. The Raman spectrum of the sample is shown in the inset of Figure 2a. The weak signal at about 648 cm^−1^ can be attributed to the Mn–O vibration [4,8]. The other two peaks at about 1366 and 1592 cm^−1^ correspond to the D-band and G-band of disordered carbon and graphitic carbon, respectively. The higher intensity of the D-band means that the amorphous carbon has more defects and can offer more lithium storage sites [8,26]. It also reveals that the major component of the obtained beaded nanofiber network is amorphous carbon with a small amount of MnO, which is in accordance with the XRD results. Moreover, an XPS investigation was performed to clarify the elements and their chemical states on the surface of the samples. All the XPS spectra were calibrated by the binding energy of C 1s at 284.6 eV. Apart from the peaks of Mn, O and C, as shown in Figure 2b, elemental N can be observed in the full scan XPS spectrum. The high-resolution XPS spectrum of C 1s shown in Figure 2c are fitted into four peaks centered at about 289.1, 286.8, 285.7 and 284.6 eV, corresponding to C–O, C–O–C, N–C and C–C bonds, respectively [4,8]. In Figure 2d, the two overlapped peaks at 400.5 and 398.9 eV in the high-resolution XPS spectrum of N 1s are assigned to the C=N (pyridinic nitrogen) and C–N (pyrrolic nitrogen) bonds, respectively [4,8,11]. As shown in Figure 2e, the Mn 2p spectrum contains two peaks at 653.2 eV and 641.5 eV, which can be respectively attributed to Mn 2p_1/2_ and Mn 2p_3/2_ configurations of Mn^2+^ in MnO [4,8,20]. Based on the full XPS spectrum, the relative content of MnO, N and C are estimated to be 11.7%, 11.7% and 76.6%, respectively.

**Figure 3:**
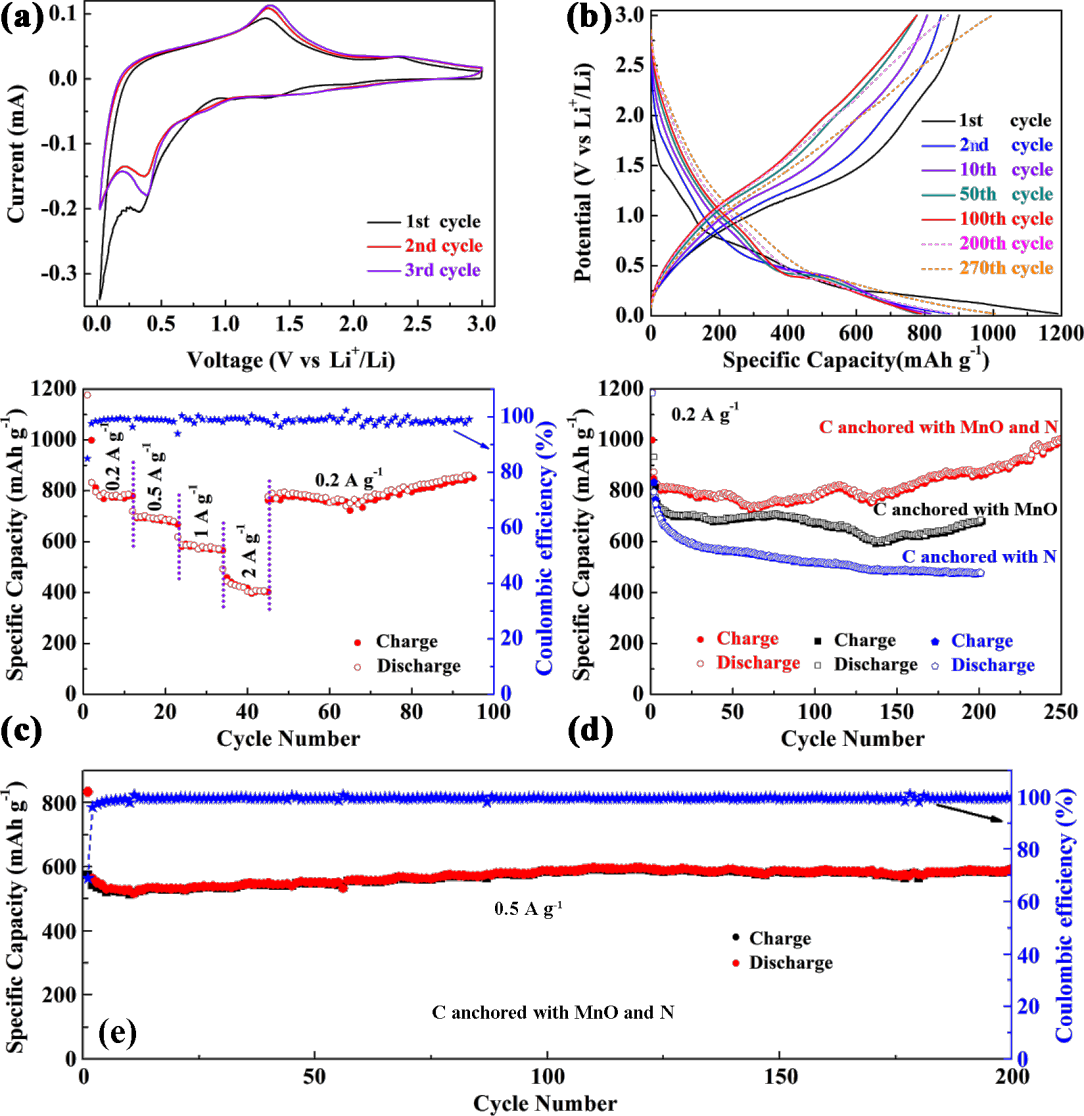
(a) Cyclic voltammetry curves, (b) galvanostatic discharge–charge profiles, (c) rate capacity, and (d, e) cycling performance of the network of spindle-like carbon nanofibers anchored with MnO and N.

The electrochemical performance of the beaded nanofiber carbon network anchored with MnO and N was investigated as a working electrode of CR2032-type coin cell with lithium foil as the counter and reference electrodes. Figure 3a shows the typical cyclic voltammetry (CV) curves of the initial three cycles. In the first discharge–charge cycle, the remarkable peak at about 0.34 V indicates that Mn^2+^ is reduced to Mn° [4,20]. In the second and third cycle, the peak slightly shifts to a higher voltage of 0.37–0.39 V, which is probably due to the improved kinetics and utilization efficiency of MnO or the microstructure change after the first lithiation process [5,7,27]. In the anodic scan, the broad peak centered at 1.31 V relates to the decomposition of Li_2_O and regeneration of MnO, which shifts to 1.33–1.36 V in the second and third cycle [8,15,20]. Additionally, the weak peak centered at 2.35 V may be ascribed to the re-oxidation of Mn^2+^ to a higher oxidation state through a new electrochemical reaction [4,7,27]. After the first cycle, the CV curves are well-overlapped, declaring a good electrochemical reversibility and structural stability of the electrode. These results are confirmed by the plateaus in the galvanostatic discharge–charge profiles shown in Figure 3b. An initial discharge capacity of 1188.6 mA h g^−1^ is obtained, which is much higher than pure MnO (755 mAh g^−1^) and three-fold higher than the theoretical values of pure carbon (372 mAh g^−1^). The initial charge capacity is 900.1 mAh g^−1^, leading to a coulombic efficiency of 75.8%. The capacity difference between the initial charge and discharge mainly owes to the electrochemically driven electrolyte degradation, which results in the formation of solid electrolyte interface (SEI) films on the surface of electrode [8,28]. In the 2nd, 10th and 50th cycle, both of the discharge and charge curves become shorter due to the ordinary capacity fading. Nevertheless, the capacity begins to increase after the 100th cycle. Many previous works have reported this similar phenomenon of the capacity first decreasing and then increasing [4,8,23,28]. For this given system, this should be mainly due to the activation of the material, the formation of a higher oxidation state of Mn and the reversibility improvement for the conversion reaction. Figure 3c shows the rate capacity tested by the stepwise increase in the current density from 0.2 to 2.0 A g^−1^. When it returns to 0.2 A g^−1^ again, the capacity recovers to 860.7 mAh g^−1^ and maintains this value until the 94th cycle without fading. Additionally, the coulombic efficiency is considerably high. To further investigate the long-term cycling performance of the electrode, the same cell with the beaded nanofiber carbon network anchored with MnO and N was tested as working electrode at 0.2 A g^−1^ and the cycling curve is shown in Figure 3d. It reveals that the capacity slightly reduces at first, then basically maintains at a constant value, and finally increases gradually upon cycling. These results are in good accordance with the galvanostatic discharge–charge tests. When the cell is tested to the 200th cycle, the reversible capacity is maintained to 875.5 mAh g^−1^, which is much larger than those of 672 and 479.1 mAh g^−1^ for the control samples of carbon anchored with MnO and carbon anchored with N, respectively. It is worth noting that the reversible capacity is as high as 1005.5 mAh g^−1^ when the cell is tested to the 250th cycle. As listed in Table 1, such a high capacity is impressive among the numerous, relevant, previous works on MnO–C-composite-based LIB anodes. To further identify the cycling performance of the networked carbon anode anchored with MnO and N, a cell was cycled at a higher current density of 0.5 A g^−1^. As shown in Figure 3e, it delivers a reversible specific capacity of 591 mAh g^−1^ after 200 cycles with a considerable coulombic efficiency of over 98.9%. Based on the above analysis, such a reliable electrochemical performance of the enhanced reversible capacity, the good rate capacity and significant cycle stability may be mainly attributed to the high theoretical capacity of MnO, the improved conductivity of carbon anchored with N, and the robust beaded nanofiber network with structural endurance that effectively alleviates the volume change of the electrode during lithium insertion–extraction.

**Table 1:** Lithium storage performance of some comparable MnO–C-composite-based anodes for lithium-ion batteries (LIBs).

Sample	Preparation strategy	LIB electrode	Capacity stability (mAh g^−1^)	Cycle number	Current density (mA g^−1^)

Coaxial MnO/N-doped C nanorods [4]	hydrothermal → polymerization → heat treatment in Ar/H_2_	slurry coating	982	100	500
MnO–C hybrid nanofibers [5]	electrospinning → heat treatment in Ar	freestanding	398	200	200
MnO–C nanofiber membranes [13]	hydrothermal → electrospinning → heat treatment in N_2_	slurry coating	655	280	500
MnO nanoparticles in C microsheets [14]	acrylic acid solution → freeze drying → heat treatment in Ar	slurry coating	798	50	100
MnO–C nanopeapods [16]	hydrothermal → solution immersion → heat treatment in Ar	slurry coating	1119	100	500
MnO–C coaxial nanowires [18]	hydrothermal → polymerization → heat treatment in N_2_	freestanding	832	100	100
MnO–C nanowires [19]	hydrothermal → polymerization → heat treatment in Ar	slurry coating	970	100	100
MnO–C core–shell nanowires [20]	hydrothermal → polymerization → heat treatment in Ar/H_2_	slurry coating	903	100	100
MnO–C coaxial nanocables [21]	hydrothermal → solution immersion → heat treatment in N_2_	slurry coating	750	150	200
MnO nanoparticles in C nanofibers [22]	solvothermal → electrospinning →heat treatment in Ar/H_2_	slurry coating	575	200	200
Nanofiber carbon network anchored with MnO and N (this work)	electrospinning → heat treatment in air and Ar	freestanding	1005	250	200
			591	200	500

## Conclusion

A novel beaded nanofiber carbon network anchored with MnO and N was fabricated via a synthesis route of electrospinning followed by preoxidation in air and postcarbonization in Ar. Compared with the beaded nanofiber carbon network anchored with MnO and anchored with N, it exhibited an enhanced reversible capacity with good rate capacity and significant recoverability when used as a LIB anode. This is mainly attributed to the high theoretical capacity and enhanced reaction kinetics of MnO, the improved conductivity of carbon anchored with N, and the robust structural endurance, effectively alleviating the problem of volume change. The work provides another credible work supporting that the transition metal oxide based carbon materials can be successfully applied for LIB electrodes.

## Experimental

### Fabrication of the carbon network

All chemicals were of analytical degree and were used without any purification. A typical procedure is as follows. Firstly, 0.45 g of manganese acetate tetrahydrate (Mn(COOH)_2_·4H_2_O), together with 0.05 g tripolycyanamide (C_3_N_3_(NH_2_)_3_) and 0.4 g PAN were dissolved in 5 mL *N*,*N*-dimethylformamide (DMF) with continuous stirring for several hours at 60 °C to obtain a homogenous spinning solution. Secondly, the obtained spinning solution was injected into a plastic syringe equipped with a metal nozzle for electrospinning. The electrospinning was conducted at a high voltage of 11 kV, a receive distance of 12 cm, and a feed rate of 0.3 mL h^−1^. Thirdly, the Mn(COOH)_2_/C_3_N_3_(NH_2_)_3_/PAN nanofiber web was deposited on an aluminum foil collector, then peeled off and kept in a tube furnace at 250 °C for 2 h in air followed by 600 °C for another 2 h in Ar. The heating and cooling rates were 5 °C min^−1^. Two control nanofiber carbon networks anchored with MnO and anchored with N were fabricated by the same procedure, but the spinning solutions were prepared by dissolving 0.5 g Mn(COOH)_2_·4H_2_O and 0.4 g PAN in 5 mL DMF, as well as dissolving 0.05 g C_3_N_3_(NH_2_)_3_ and 0.4 g PAN in another 5 mL DMF, respectively.

### Characterization of the carbon network

The microstructural and morphological characterization of the as-fabricated carbon networks were conducted by field-emission scanning electron microscopy (FE-SEM, S-4800, Hitachi) and transmission electron microscopy (TEM, FEI, Tecnai G^2^ F30). X-ray powder diffraction (XRD, X’Pert Pro, Philips), Raman spectroscopy (Jobin–Yvon Horiba, HR800) and X-ray photoelectron spectroscopy (XPS, Kratos, Axis Ultra^DLD^) analyses were carried out to determine the chemical structure and composition of the samples.

### Electrochemical characterization of the carbon network

The electrochemical properties of the carbon networks were studied with a CR2032-type coin cell. The details of the cell assembly can be found in previous works [11,29]. The galvanostatic discharge–charge cycling and rate tests of the cells were measured using a Neware BTS-610 multichannel battery tester at room temperature. Cyclic voltammetry measurements were performed over a potential window of 0.02–3.00 V vs Li/Li^+^ at a scanning rate of 0.1 mV s^−1^ with a CHI-660C electrochemical workstation.
